# Sharing primary percutaneous coronary intervention care: first experiences with South Limburg ST-elevation myocardial infarction network

**DOI:** 10.1007/s12471-021-01541-2

**Published:** 2021-02-03

**Authors:** A. Lux, J. Vainer, R. A. L. J. Theunissen, L. F. Veenstra, I. Kasperski, B. C. G. Gho, M. Stein, M. Ilhan, A. W. Ruiters, P. J. C. Winkler, A. van Beurden, W. Dohmen, S. Rasoul, A. W. J. van ’t Hof, South Limburg Interventional Cardiology Group, the Netherlands, A. Lux, J. Vainer, R. A. L. J. Theunissen, L. F. Veenstra, I. Kasperski, B. C. G. Gho, M. Stein, M. Ilhan, A. W. Ruiters, P. J. C. Winkler, S. Rasoul, A. W. J. van ’t Hof

**Affiliations:** 1 Faculty of Health, Medicine and Life Sciences, Maastricht University, Maastricht, The Netherlands; 2 Heart+Vascular Center, Maastricht University Medical Centre, Maastricht, The Netherlands; 3 Department of Cardiology, Zuyderland Medical Centre, Heerlen, The Netherlands; 4 Department of Medical Management, Municipal Health Services South Limburg, Heerlen, The Netherlands

**Keywords:** Primary PCI network, Regional care, Quality indicator

## Abstract

**Background:**

In the region of South Limburg, the Netherlands, a shared ST-elevation myocardial infarction (STEMI) networking system (SLIM network) was implemented. During out-of-office hours, two percutaneous coronary intervention (PCI) centres—Maastricht University Medical Centre and Zuyderland Medical Centre—are supported by the same interventional cardiologist. The aim of this study was to analyse performance indicators within this network and to compare them with contemporary European Society of Cardiology guidelines.

**Methods:**

Key time indicators for an all-comer STEMI population were registered by the emergency medical service and the PCI centres. The time measurements showed a non-Gaussian distribution; they are presented as median with 25th and 75th percentiles.

**Results:**

Between 1 February 2018 and 31 March 2019, a total of 570 STEMI patients were admitted to the participating centres. The total system delay (from emergency call to needle time) was 65 min (53–77), with a prehospital system delay of 40 min (34–47) and a door-to-needle time of 22 min (15–34). Compared with in-office hours, out-of-office hours significantly lengthened system delays (55 (47–66) vs 70 min (62–81), *p* < 0.001), emergency medical service transport times (29 (24–34) vs 35 min (29–40), *p* < 0.001) and door-to-needle times (17 (14–26) vs 26 min (18–37), *p* < 0.001).

**Conclusions:**

With its effective patient pathway management, the SLIM network was able to meet the quality criteria set by contemporary European revascularisation guidelines.

## What’s new?

- The two percutaneous coronary intervention (PCI) centres of South Limburg, the Netherlands have established a novel shared ST-elevation myocardial infarction (STEMI) networking system (SLIM network), in which one on-call interventional cardiologist effectively serves both regional centres.
- In this study, the SLIM network’s dynamic patient pathway management kept regional time delays within the limits set by contemporary European revascularisation guidelines.
- When sharing an interventional cardiologist, timely site activation and effective communication are essential to achieve timely reperfusion.

## Introduction

Regional networks of hospitals, which are connected by emergency medical services (EMSs), are essential to treat ST-elevation myocardial infarction (STEMI) [1–3]. These networks improve clinical outcomes by shortening the time until coronary artery reperfusion [4–6]. Experienced centres and clear patient pathways are critical for these networks; thus, guidelines discourage the implementation of rotation models [1]. Still, some densely populated regions have proven that, under special circumstances, shared systems are effective and improve quality indicators [3, 7]. The region of South Limburg, the Netherlands has taken this rotation model a step further. To our knowledge, our network is the first to provide rotation of STEMI care at two locations, supported by the same team of interventional cardiologists.

Herein, we present the performance of our network in the light of contemporary revascularisation guidelines. With this analysis, we also aimed at a better understanding of the prevalence and impact of simultaneous admissions at two locations.

## Methods

### Organisational and regional characteristics

**Fig. 1:**
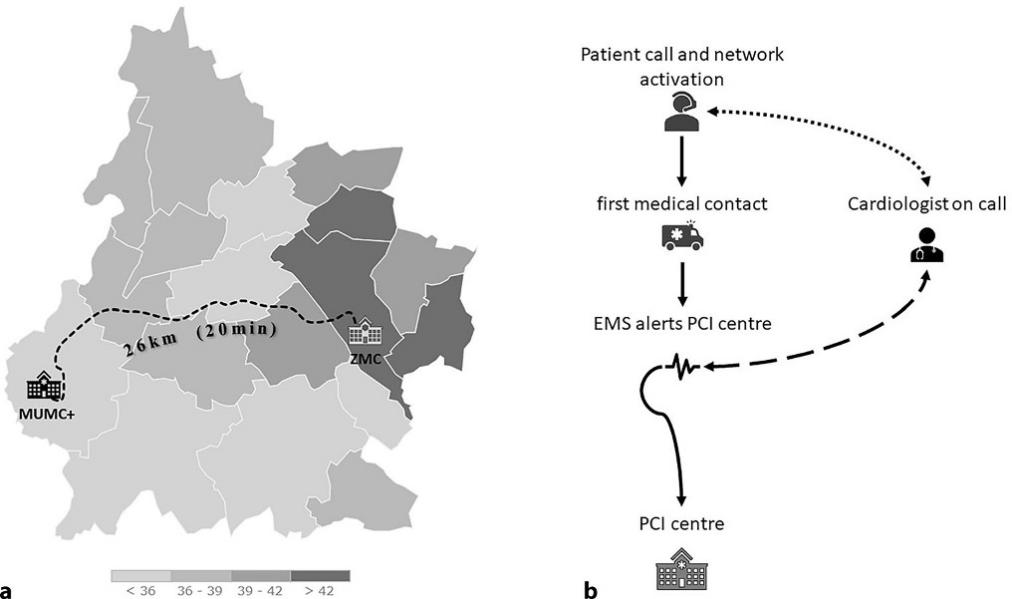
Region and structure of South Limburg STEMI network. **a** Local communities are demarcated with *white lines*; *shades* represent portion of individuals (<36% through > 42%) who are > 19 years and have at least one chronic medical condition (source: www.gezondheidsatlaszl.nl). *Dashed line* is quickest route between two percutaneous coronary intervention (PCI) centres. **b** Regional chain of emergency care. Interventional cardiologist shares his or her current location with emergency medical service (EMS) call central and reads electrocardiogram for PCI centre (*dotted and dashed lines*). (*MUMC+* Maastricht University Medical Centre, *ZMC* Zuyderland Medical Centre)

South Limburg is one of the areas with the highest–cardiovascular risk population in the Netherlands. In this region, there is a high heterogeneity in health profiles due to the relatively healthy population in the area of Maastricht compared with the industrial areas of Heerlen (e.g. 266 vs 320 cardiovascular deaths per 100,000 inhabitants in 2018, respectively) [8, 9]. The two primary percutaneous coronary intervention (PCI) centres located in this region—Maastricht University Hospital (MUMC+) and Zuyderland Medical Centre in Heerlen (ZMC)—are responsible for approximately 600,000 inhabitants; the centres are 26 kilometres apart (Fig. 1; [10]). In 2019, approximately 3100 PCI’s, including fractional flow reserve, were performed at these two centres. Our centres employ nine full-time interventional cardiologists, five of whom work at both locations on a regular basis. During the observed period, these interventional cardiologists either performed the primary PCI themselves or supervised their fellows on site.

In 2016, a weekly rotation of regional PCI centres and interventional cardiologists was introduced. During the weekends and between 17:00 and 08:00 (i.e. out-of-office-hours), STEMI patients are transported to the location on call (primary location), while in-hospital patients are treated in their own centre. If the interventional cardiologist is needed at the secondary site, the EMS is informed and patient pathways are redirected to the secondary site until further notice from the interventional cardiologist. At both locations, a team of catheterisation laboratory (cath lab) personnel is available, while surgical backup is provided by the MUMC+ (Fig. 1).

### Data collection

This paper focuses on structural and performance measures of consecutive STEMI admissions between 1 February 2018 and 31 March 2019. Analyses were performed as part of the regular regional quality control process. The regional EMS was responsible for the registration of system activation (112 call), first medical contact and centre activation. Patient arrival times were registered both by the EMS and the hospitals. Needle times were registered at the cath labs. PCI centres were also responsible for registering personnel travel times. Accidental communication errors within the chain of emergency care were not registered. Individuals performing the registration were not involved in the quality control and analysis of the dataset. Data were pooled in a central database. Missing values were checked in the available source documents and if possible, recovered by two members of our team.

Based on the aforementioned indicators, the following time intervals were calculated: system delay (EMS alert to needle time), prehospital delay (EMS alert to arrival at PCI centre), EMS transport time (EMS arrival at patient to EMS arrival at PCI centre) and door-to-needle time (EMS arrival at PCI centre to first puncture at cath lab).

### Statistical analysis

Statistical analysis was performed with IBM SPSS Statistics for Windows, Version 25.0 (IBM Corp., Armonk, NY, USA). Variables with a non-Gaussian distribution are presented as median with 25th and 75th percentiles and categorical variables are presented in both absolute numbers and percentages. Numerical variables were compared with the Mann-Whitney U test, while the chi-squared test was used to compare categorical variables. Data were stratified based on the time of the procedure (in-office vs out-of-office hours). The number of missing cases was similar for in-office and out-of-office hours and were excluded test-by-test.

Binary logistic regression analysis was used to evaluate the confounding effect of location and ongoing procedures on clinically significant time delays. The effect of timely site activation on door-to-needle time during out-of-office hours was analysed with a bivariate correlation test. Cases were excluded pairwise, and the Pearson’s correlation coefficient was calculated. A *p*-value < 0.05 was considered significant.

## Results

### Completeness of registration

Traditional indicators, such as EMS alert (112 call) and needle time, had the highest registration rates (>80%; Tab. 1). Personnel-related time stamps and intermediate moments, such as alerting the primary PCI centre or arrival at the emergency department, were less frequently recorded (<67%; Tab. 1).

**Table 1:** Registered key time indicators

Key time indicator	*n*/*N* (%)
Alert EMS	477/571 (84)
EMS arrival by patient	461/571 (81)
EMS arrival at pPCI centre	450/571 (79)
Alert pPCI centre	302/571 (53)
Arrival at ER	383/571 (67)
Arrival at cath lab	465/571 (81)
Needle time	455/571 (80)
Alert interventional cardiologist	189/345 (55)^a^
Arrival interventional cardiologist at cath lab	188/345 (55)^b^

*EMS* emergency medical service, *pPCI* primary percutaneous coronary intervention, *ER* emergency room, referring to emergency departments and cardiac emergency departments of participating centres, *cath lab* heart catheterisation laboratory

^a^In 32 cases, at least one technician was already present

^b^In 30 cases, at least one technician was already present

### Number and distribution of STEMI patients

During the screened period, 608 of the registered patients had an initial diagnosis of STEMI; for 570 of them (94%), time registrations and a verified diagnosis after arrival at the primary PCI centre were available. These 570 patients were evenly distributed among the MUMC+ (47%) and the ZMC (53%). More patients were treated during out-of-office hours than during in-office hours (60% vs 40%, respectively). Patient inflow during in-office and out-of-office hours was less balanced in the MUMC+ (33% vs 67%) than in the ZMC (45% vs 55%) (Tab. 2).

**Table 2:** Admissions for ST-elevation myocardial infarction per location and time frame

	*n* (%)		
Time frame	MUMC+	ZMC	Total
In-offfice hours	90 (33)^a^	133 (45)^a^	223 (40)
Out-of-office hours	179 (67)^a^	168 (55)^a^	347 (60)
Total	269 (47)^b^	301 (53)^b^	

*MUMC+* Maastricht University Medical Centre, *ZMC* Zuyderland Medical Centre

^a^Percentage of total number of patients per location

^b^Percentage of total number of patients included in analysis

### System delay and door-to-needle time within the SLIM network

**Fig. 2:**
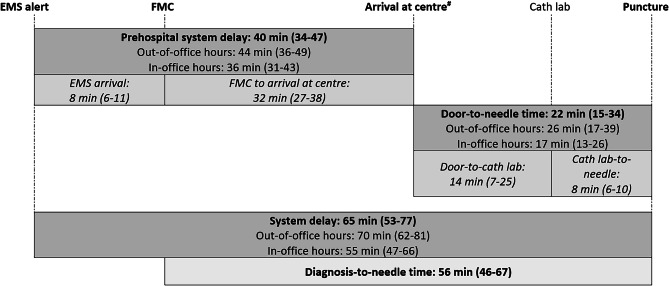
Time delay within South Limburg ST-elevation myocardial infarction network. Data are presented as median with 25th and 75th percentiles. #Arrival times were registered by both emergency medical service (EMS) and percutaneous coronary intervention centre. For current analysis, EMS registration was prioritised. *FMC* first medical contact, *Cath lab* heart catheterisation laboratory

**Fig. 3:**
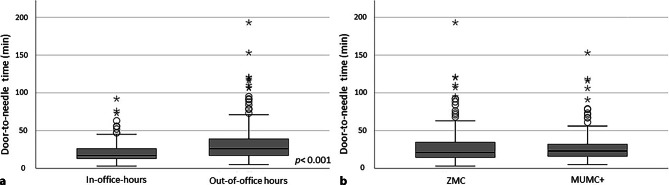
Door-to-needle time (in minutes). **a** In-office versus out-of-office hours. **b** Zuyderland Medical Centre in Heerlen (ZMC) versus Maastricht University Medical Centre (MUMC+). Variables were compared with Mann-Whitney U test and are presented as median with 25th and 75th percentiles. Outliers are marked with *circles* (‘out’) or *stars* (‘far out’)

The median regional total system delay (EMS activation to needle time) was 65 min (53–77), with a prehospital system delay of 40 min (34–47) and a door-to-needle time of 22 min (15–34) (Figs. 2 and 3). First medical contact-to-needle time was 56 min (46–67). Of the 353 patients for whom a first medical contact-to-needle time was available, 342 (96%) were treated within 120 min. Compared with in-office hours, out-of-office hours had a negative impact on system delay (70 (62–81) vs 55 min (47–66), *p* < 0.001) and door-to-needle time (26 (18–37) vs 17 min (14–26), *p* < 0.001) (Fig. 3a).

### Occupied cath labs and patient delays

Within this cohort, 16 patients presented during an ongoing procedure (elective or acute). These 16 patients were evenly distributed among the locations. During out-of-office hours, 11 patients encountered delays due to an ongoing procedure; 2 of these delays were related to a procedure at the other location. Delays due to ongoing (elective or acute) procedures prolonged door-to-needle time (21 (15–32) vs 63 min (39–92), *p* < 0.001) and system delay (65 (53–75) vs 91 min (73–114), *p* < 0.001). Ongoing procedures were a risk factor for prolonged door-to-needle time (>30 min, odds ratio (OR) 17 during in-office hours, *p* = 0.008; OR 13 during out-of-office hours, *p* < 0.001) and on-call system delay (>120 min, OR 11, *p* = 0.003). These indicators were similar for both locations and were not influenced by parallel admissions.

### Chain of emergency care and arrival times during out-of-office hours

First medical contact took place 8 min (6–11) after EMS alert. First medical contact-to-diagnosis time was not available. The EMS needed 15 min (11–22) to make an initial diagnosis, prepare the patient and contact the local PCI centre. After alerting the PCI centre, it took the EMS approximately 15–22 min to arrive at the destination (median time for the region: 18 min (10–26); ZMC: 15 min (6–23); MUMC+: 22 min (16–30)). Interventional cardiologists had a median travel time of 15 min (13–31). We found a significant correlation between door-to-needle time delay and delayed alerting of PCI centres (r = −0.357, *n* = 148, *p* < 0.001).

### Secondary site admission

During out-of-office hours, the secondary site admitted 32 STEMI patients (MUMC+: 22/168; ZMC: 10/179). Two of the secondary site admissions took place within a 1-hour window and one walk-in STEMI patient was registered at the secondary site.

## Discussion

We set up a novel primary PCI network (SLIM network) and showed that, under special circumstances and with proper logistics, PCI centres can effectively share their interventional cardiologists and meet the quality criteria set by contemporary European Society of Cardiology (ESC) guidelines [1].

The quality and efficacy of STEMI systems are usually measured with key time indicators. They are patient- and system-related delays of STEMI care; the latter consists of prehospital delays (EMS and transportation delays) and in-hospital delays [1, 6]. These delays are important, as they correlate with short- and long-term outcomes of STEMI patients [5, 6]. To keep these delays as short as possible, the ESC emphasises the importance of transparent and effective STEMI networks and strongly recommends 24/7 on-call systems for primary PCI centres [1].

Regional peculiarities of geography, population density and hospital logistics have already led to unique solutions, such as centre rotations in the Vienna and Budapest regions [3, 7, 11, 12]. There are, however, significant differences between these networks and the SLIM network. Where both Vienna and Budapest are densely populated metropolitan areas with at least five PCI centres operating in their rotations, South-Limburg is an average-sized region with two PCI centres [7, 12]. Another important difference is the sharing of interventional cardiologists between the two Dutch centres. While the Austrian and Hungarian centres only work with their own interventional cardiologists, the MUMC+ and ZMC optimise their primary PCI rotations by sharing staff members.

Since more admissions occurred during out-of-office hours, we believe that the SLIM network evenly distributed the number of acute admissions between the regional centres (Tab. 2). This should lead to a balanced regional operator experience, with all interventional cardiologists easily meeting the national accreditation criteria (>30 PCIs/year). Since being on-call disrupts everyday life regardless of the actual number of calls, our colleagues also preferred the ‘rare and intense’ shifts over ‘balanced and mild’ rotations.

There are several options to measure the efficacy of STEMI systems, as proven by publications discussing changes in treatment availability, mortality and time delays [7, 11, 12]. Since primary PCI networks are well established in the Netherlands, it was less important to analyse treatment availability in the current study [3]. With synchronised medical protocols, shared personnel and matched PCI equipment, regional time delays remain the most relevant quality indicator as they correlate directly with mortality rates [5, 6]. Therefore, even though there are well-known difficulties with data registration, regional time delays were chosen to demonstrate the efficacy of the SLIM model [13].

Despite the lack of contemporary evidence, European guidelines have left the 120-minute time window for initial decision-making (PCI vs fibrinolysis) unchanged and still advise a maximum of 90 min for the diagnosis-to-reperfusion time [1]. In the present analysis, both the diagnosis-to-needle time (56 min (46–67)) and the system delay (EMS alert to needle time: 65 min (53–77)) were shorter in the SLIM network (Fig. 2) than the advised 90 min and were clearly below the 108–120 min threshold for increased mortality rates [1, 5, 6]. Although we analysed an ‘all-comer’ STEMI population, the median arrival time was close to 60 min, the time point when the next significant step down in mortality rates is expected [5, 6, 13].

Door-to-needle and door-to-balloon (reperfusion) times are an integral part of system delay and have a significant correlation with in-hospital and long-term mortality rates [14]. Still, the ESC does not offer clear recommendations on the maximum duration of these times. Presumably, as long as the diagnosis-to-reperfusion time recommendations of 90–120 min are respected, door-to-needle time remains a secondary indicator for STEMI networks [1]. Nevertheless, door-to-needle and door-to-balloon times are almost entirely PCI centre–dependent and are therefore important local quality indicators [13].

Although regional system delays and door-to-needle times complied with the contemporary revascularisation guidelines, we also noticed that ongoing procedures, out-of-office hours and delayed site activation had a negative influence on these key time indicators. As for the ongoing procedures, cath lab ‘jams’ were predominantly seen when patients were already being treated at the same location. Simultaneous site activation was rare and had no effect on the median delays.

Time delays are not only influenced by patient- and EMS-related factors, but also by cath lab availability. As customary in the Netherlands, our interventional cardiologists are not required to remain in the hospital during out-of-office hours, but they must be ‘ready to reperfuse’ within 30 min. Our interventional cardiologists live in different areas of South Limburg and have an estimated travel time of 5–25 min and a measured arrival time of 15 min (13–31). Regional EMS transport times resonate well with both the 30-minute deadline and the staff travel times. Our data also suggest that a delay in alerting the PCI centre, and consequently its personnel, could add several minutes to the local door-to-needle times. Optimising patient pathways based on the medical team’s actual location and availability is also essential to avoid unnecessary delays caused by travelling interventional cardiologists. Some medical emergencies and conditions (e.g. out-of-hospital cardiac arrest, cardiogenic shock and ruling out of potentially life-threatening secondary diagnoses) are difficult to generalise, but have contributed to median delays and outliers [13].

### Limitations

The SLIM network model is still evolving and our results reflect the actual status of collaboration. Based on the available data, potential changes in quality of care relative to the system prior to 2016 or the impact of communication errors could not be evaluated. The MUMC+ and ZMC are autonomous entities: data registration and reporting methods are left to their own discretion and are not yet standardised. Times were registered by personnel actively involved in patient care. Other acute admissions or urgent procedures (e.g. for patients with non-ST-elevation acute coronary syndrome) were not included in the analysis, but these could have caused significant delays. In one or two cases, a second interventional cardiologist was involved in patient care, but these involvements were not properly registered and could therefore not be analysed.

## Conclusion

With its effective patient pathway management, the SLIM network was able to meet the time delay requirements set by contemporary European revascularisation guidelines.
